# Enterovirus virus-like-particle and inactivated poliovirus vaccines do not elicit substantive cross-reactive antibody responses

**DOI:** 10.1371/journal.ppat.1012159

**Published:** 2024-04-25

**Authors:** Daniel L. Moss, Alden C. Paine, Peter W. Krug, Masaru Kanekiyo, Tracy J. Ruckwardt

**Affiliations:** Vaccine Research Center, National Institute of Allergy and Infectious Diseases, National Institutes of Health, Bethesda, Maryland, United States of America; National University of Singapore Yong Loo Lin School of Medicine, SINGAPORE

## Abstract

Human enteroviruses are the most common human pathogen with over 300 distinct genotypes. Previous work with poliovirus has suggested that it is possible to generate antibody responses in humans and animals that can recognize members of multiple enterovirus species. However, cross protective immunity across multiple enteroviruses is not observed epidemiologically in humans. Here we investigated whether immunization of mice or baboons with inactivated poliovirus or enterovirus virus-like-particles (VLPs) vaccines generates antibody responses that can recognize enterovirus D68 or A71. We found that mice only generated antibodies specific for the antigen they were immunized with, and repeated immunization failed to generate cross-reactive antibody responses as measured by both ELISA and neutralization assay. Immunization of baboons with IPV failed to generate neutralizing antibody responses against enterovirus D68 or A71. These results suggest that a multivalent approach to enterovirus vaccination is necessary to protect against enterovirus disease in vulnerable populations.

## Author summary

Enteroviruses are common human pathogens with the potential to cause severe disease and life-long paralysis and even death. The ability for enterovirus infection and vaccination to generate cross-reactive antibody responses in humans and animal models is not completely understood. Understanding whether cross-reactive antibody responses can be elicited is important for vaccine development strategies to counter existing and emerging enterovirus threats to human health and is part of a broader pandemic preparedness program. This study evaluated the generation of cross-reactive enterovirus antibodies in animals after vaccination with inactivated poliovirus vaccine or enterovirus virus-like-particles to inform vaccine development for emerging and re-emerging enteroviruses.

## Introduction

Human enteroviruses belong to the *Picornaviridae* family which contains polioviruses and nonpolio enteroviruses (NPEVs) such as enterovirus D68 (EV-D68) and enterovirus A71 (EV-A71). Enteroviruses are the most common human pathogen with over 300 different genotypes. While most are mild, enterovirus infections can cause a range of disease outcomes such as a paralytic syndrome known as acute flaccid myelitis (AFM), aseptic meningitis, neonatal sepsis, hand foot and mouth disease, encephalitis, gastroenteritis and severe respiratory disease [1]. In the mid-20^th^ century poliovirus outbreaks reached pandemic proportions in Europe and North America leading to permanent paralysis or death for tens of thousands of children in the United States alone [2]. The introduction of the oral and later inactivated poliovirus vaccine (IPV) lead to eradication in much of the world. The time period from 1988 to 2014 saw a reduction from 125 endemic countries to only 3 and from 350,000 cases to only 359 [3]. EV-A71 and coxsackieviruses in the enterovirus A species cause hand, foot, and mouth disease, and can progress to meningitis, encephalitis, and AFM, a major source of childhood morbidity and mortality throughout east and southeast Asia [4]. Neurological complications due to EV-A71 infection prompted the development of an inactivated virus vaccine in China in 2010 that has been shown to be highly effective at reducing hand foot and mouth disease burden and neurologic complications [5–8]. The toll of enteroviruses on human health and the effectiveness of vaccines to mitigate disease caused by enteroviruses supports the continued development of countermeasures against these pathogens.

EV-D68 is a respiratory enterovirus that has been associated with severe respiratory disease and AFM outbreaks in the United States and Europe in 2014, 2016 and 2018 [9–15]. EV-D68 outbreaks were also observed in the United States in 2022 but were not associated with an increase in AFM diagnoses [16]. Currently there are no approved vaccines or antiviral treatments for EV-D68. Treatment consists of supportive care for respiratory disease and rehabilitation for AFM. Given the prior successes of poliovirus vaccines, the development of an EV-D68 vaccine is a promising path for reducing disease burden and AFM caused by this re-emerging virus. We have recently described an EV-D68 virus-like-particle (VLP) vaccine that induces potent neutralizing antibodies in mice and nonhuman primates that can protect mice from infection in an EV-D68 respiratory infection model [17].

Enteroviruses contain a single positive-strand RNA genome that encodes the viral polyprotein, which is processed into the nonstructural and structural viral proteins with the latter forming the characteristic icosahedral capsid possessing five-, three- and two-fold symmetry [18]. Structural proteins VP1-4 form protomers that assemble into pentamers, and the capsid is formed by twelve pentamers. For polioviruses, EV-A71, and EV-D68, antigenic sites of vulnerability have been described at the five- and three-fold axes of symmetry and neutralizing monoclonal antibodies have been identified in both mice and humans [19–24]. Receptor binding for polioviruses and EV-A71 occurs at the five-fold axis of symmetry which is comprised primarily of VP1 protein [25,26]. The receptor(s) for EV-D68 are still debated, but binding is believed to also occur at the five-fold axis [27]. Receptor binding dictates enterovirus tissue tropism and is a major source of variability across enteroviruses and therefore a difficult target for cross-reactive antibody responses. The three-fold axis of symmetry is comprised of VP2 and VP3 proteins and is important for structural changes involved with capsid uncoating and genome release and may be a cause of variable acid-sensitivity observed among some enterovirus species [25,28]. However, it is unclear if and how immunization with inactivated virus or VLP vaccines induce cross-reactive antibodies that can bind to, and potentially neutralize, heterologous enteroviruses.

Cross-reactive enterovirus responses have been studied in animals. Antisera from type 3 polio VP1 peptide-immunized rabbits bound to other enterovirus species suggesting that it is possible to generate these cross-reactive antibodies in an animal model [29]. In another study, mice were immunized with recombinant enterovirus VP1 proteins, and a set of four monoclonal antibodies were isolated that, when combined, recognize 41 different enteroviruses by indirect fluorescent antibody assay [30]. Whether these antibodies possess neutralizing activity was not reported. Similarly, a mouse monoclonal antibody against EV-A71 VP1 protein can bind coxsackievirus-A16 VP1 by western blot but it is not clear if it is neutralizing [30]. A more recent study described the generation of cross-reactive polyclonal sera in mice immunized with poliovirus or a variety of other enteroviruses. Pooled sera from mice immunized with type 1 poliovirus was capable of neutralizing multiple EV-D68 isolates, and detectable neutralization of type 1 polio was present in sera pooled from mice repeatedly immunized with EV-D68 or EV-D94 [31].

Cross-reactive enterovirus antibodies have also been identified in humans, but unknown exposure history is a significant barrier to understanding whether these responses are bona fide or were elicited by an asymptomatic or mild infection with a homologous or highly similar virus. Polyclonal serum from patients with culture-confirmed enteroviral infections can react against synthetic poliovirus peptides suggesting that it is possible to generate cross-reactive enterovirus antibodies by infection [32]. Conserved portions of peptides from type 1 polio VP1 protein have been shown to react with serum from humans with aseptic meningitis caused by different enterovirus infections [33]. However, in both studies the poliovirus vaccination status of these subjects is not discussed. A monoclonal antibody has been described that can neutralize polio types 1 and 2 and bind to, but not neutralize, type 3 polio [34]. More recently a human monoclonal antibody was isolated and shown to neutralize all three poliovirus serotypes by binding to the receptor binding site [21,35]. The impact of these types of antibodies, which may represent a minority of the polyclonal response to infection, is unclear. Importantly, monoclonal antibodies that neutralize multiple different enterovirus species have not been described. Furthermore, whether cross-species antibodies can be elicited from typical human enterovirus vaccine regimens is unknown.

We aimed to determine if cross-reactive antibody responses could be elicited following three monovalent vaccination regimens with inactivated poliovirus vaccine, EV-D68 VLP, or EV-A71 VLPs in animals. We focused on poliovirus due to high vaccine uptake in the western world, and EV-D68 and EV-A71 which are more recently associated with AFM development [36]. In addition to the mouse studies, we also obtained serum samples from baboons that received three doses of IPV to test for the presence of EV-D68 or EV-A71 specific antibodies. We found that IPV or VLP immunization was not sufficient to generate cross-species reactive enterovirus binding or neutralizing antibodies. Our data suggest that vaccine development strategies should consider multivalent formulations to protect against diverse enteroviruses associated with human disease.

## Results

### Surface exposed residues of human enterovirus capsid proteins are highly diverse

**Fig 1:**
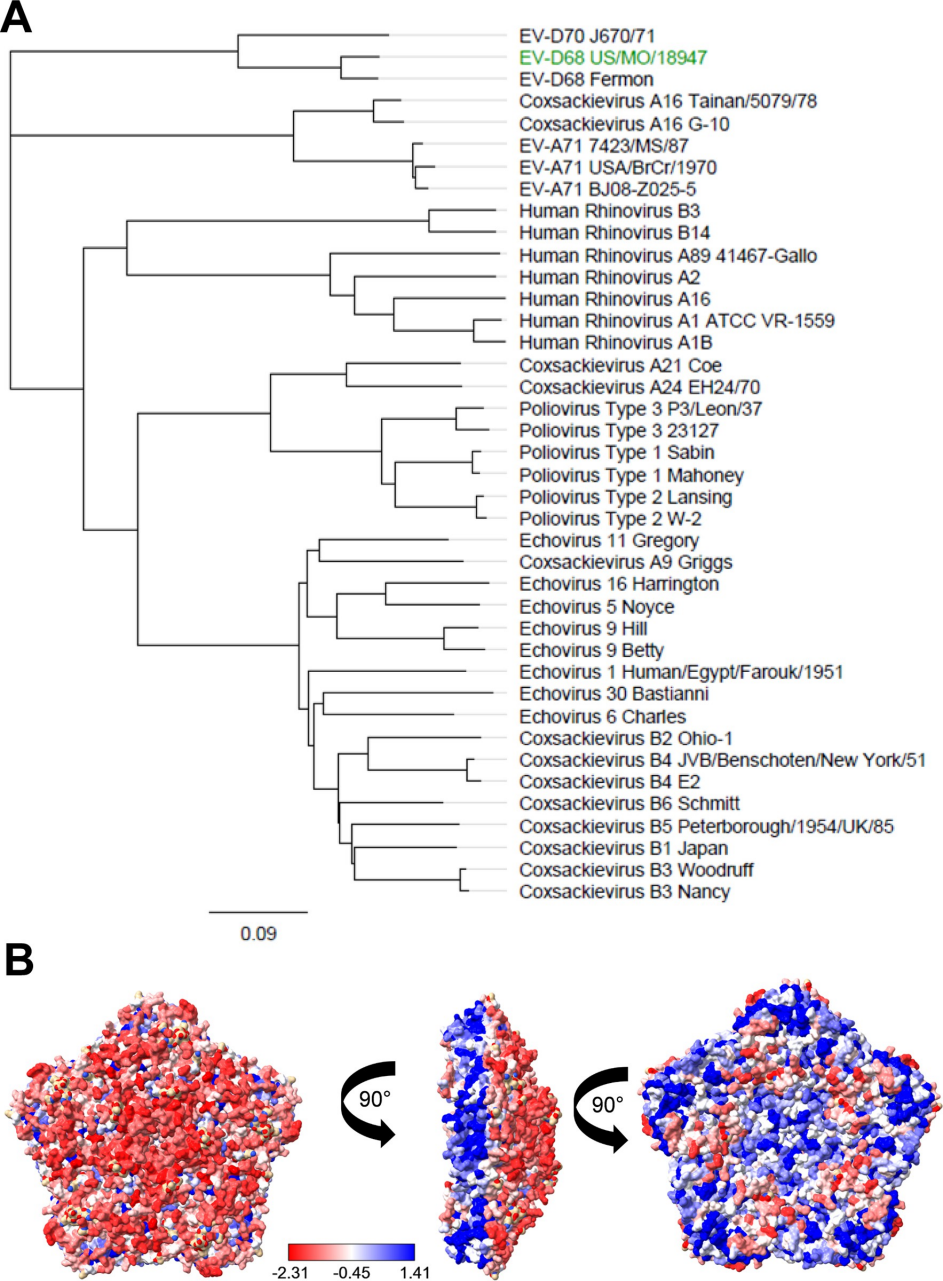
Enterovirus Capsid Conservation Across Various Species. A. Phylogenetic tree generated using Geneious Prime software and the capsid protein multiple sequence alignment. The reference sequence was set to EV-D68 US/MO/18947 (in green) which was used to generate the molecular structure below. B. Molecular structure of Enterovirus D68 capsid pentamer (PDB: 6CRR) colored based on conservation score calculated by ChimeraX software using a multiple sequence alignment of 40 different enterovirus capsid sequences. Residues range from red to white to blue from least conserved to most conserved across the alignment as calculated by the AL2CO algorithm.

We began our investigation by examining sequence conservation in the capsid proteins of different human enterovirus species to explore the potential for conserved antigenicity among enteroviruses. We used the capsid sequence for an EV-D68 2014 isolate (EV-D68 US/MO/18947) for which there is a corresponding molecular structure (PDB: 6CRR) to conduct a BLAST search of the Uniprot protein database to identify capsid sequences of enteroviruses similar to EV-D68 [28]. We selected the top 40 capsid sequences and performed a multiple sequence alignment and generated a phylogenetic tree (Fig 1A). The major branches of the phylogenetic tree consisted of enterovirus D and A species as well as human rhinoviruses and enterovirus C species, represented primarily by poliovirus, as well as a major branch containing echoviruses and coxsackie B viruses. Using the multiple sequence alignment, we generated a conservation score and colored the residues within the capsid pentamer based on computed sequence conservation (Fig 1B) [37]. The most conserved residues are in the interior of the pentamer and are primarily located along the interface where pentamers meet to form the capsid. The least conserved residues were located on the exterior or the pentamer face where receptor binding and antigenic recognition by antibodies occurs. Enteroviruses exhibit a wide range of capsid protein sequence diversity, and diversity is enriched in surface exposed residues, suggesting that eliciting broad or cross-species protection to conformational capsid epitopes will be challenging.

### Inactivated enterovirus and VLP vaccine preparations contain contaminating host-cell proteins

**Fig 2:**
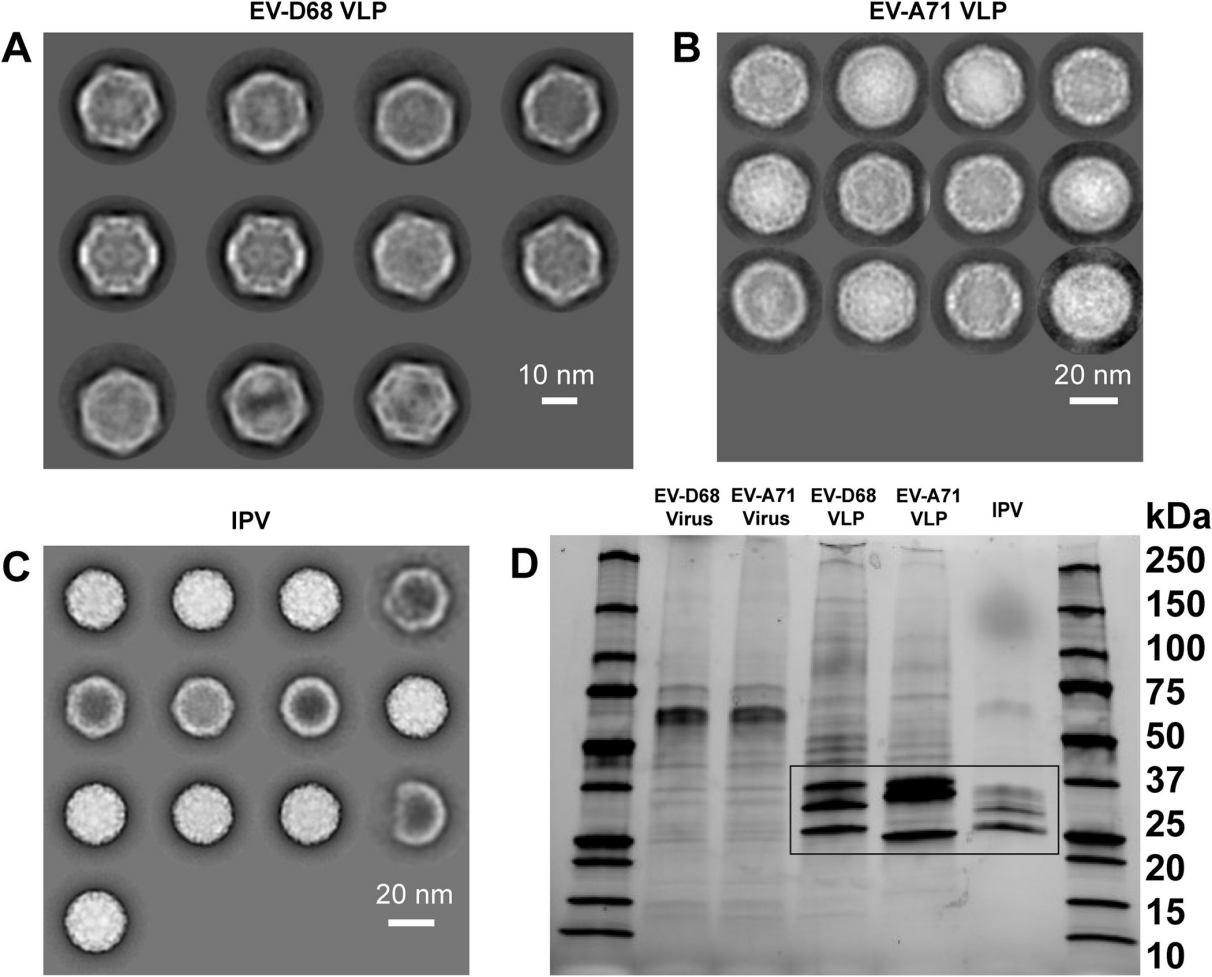
Enterovirus and enterovirus-like-particle preparations contain contaminating host-cell proteins. A-C. Transmission electron microscopy of purified EV-D68 VLPs (A), purified EV-A71 VLPs (B) and concentrated IPV particles (C). D. SDS-Polyacrylamide gel showing the protein content of enterovirus and VLP preps. The identity of each sample is listed above the lane. Bands representing VLP proteins or IPV proteins are highlighted by a black box.

EV-D68 and EV-A71 VLPs used in this study, along with the commercial IPV, were all shown to contain symmetric, well-formed particles when analyzed by transmission electron microscopy (Fig 2A–2C). EV-D68 and EV-A71 viral stocks propagated in RD cells containing 1% fetal bovine serum (FBS) contain some contaminating host-cell proteins (Fig 2D). In these samples viral proteins are not abundant enough to be readily distinguished from host-cell and bovine serum proteins present in the samples. VLPs prepared using human 293 cells and purified by density ultracentrifugation can contain a substantial amount of host-cell proteins (Fig 2D). Inactivated poliovirus vaccine (IPOL) is prepared in Vero cells and despite more advanced processing than our NPEV virus-like-particles, trace amounts of contaminating host-cell proteins in highly concentrated preparations can be detected by gel electrophoresis (Fig 2D). Therefore, based on standard purification methods, preparations containing mostly intact VLP were obtained, but contain varying degrees of host cell contaminants. These contaminants present a fundamental challenge for evaluating enterovirus antibody responses after vaccination because they can elicit antibodies in mice that appear to be cross-reactive for alternative enterovirus vaccine preparations made in the same cell type.

### Immunization of mice with NPEV VLPs or IPV does not generate cross-reactive enterovirus antibody responses

**Fig 3:**
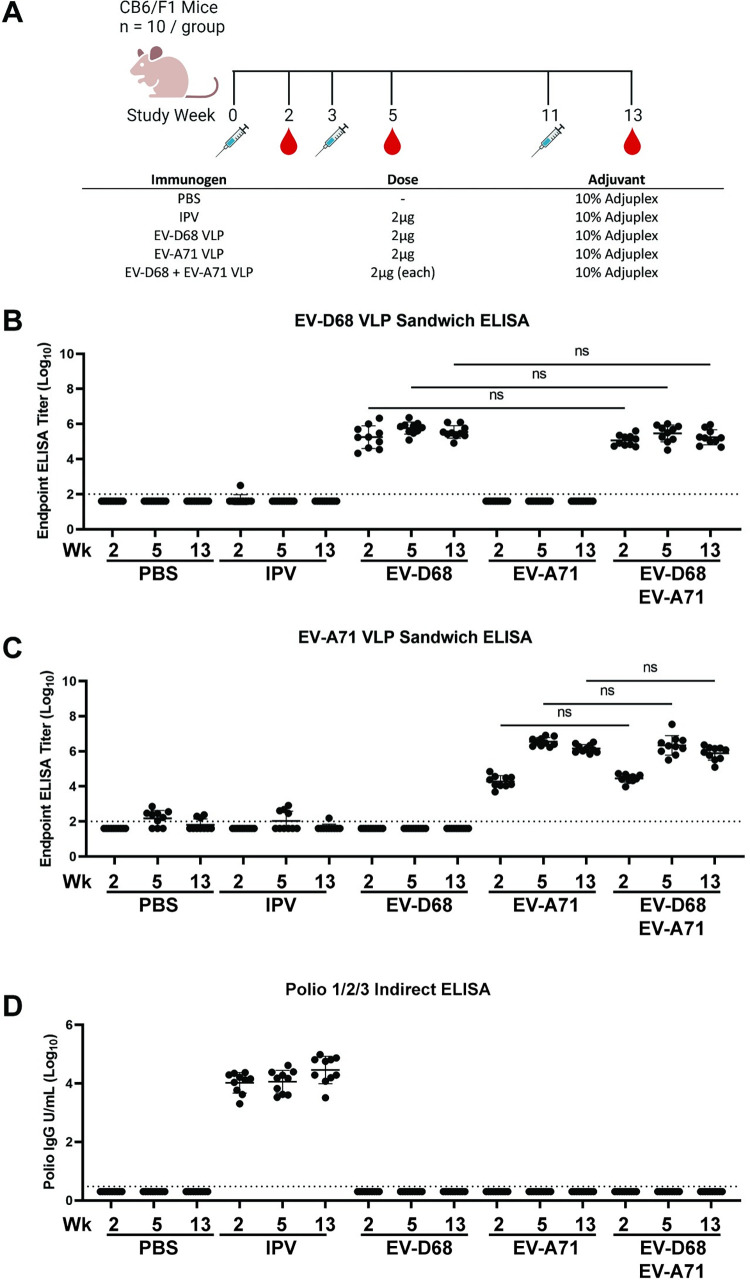
Immunization of mice with enterovirus VLPs or IPV fails to generate detectable cross-reactive binding antibody responses using a sandwich ELISA. A. Study design showing immunization and blood sampling schedule along with immunization groups. B. Sandwich ELISA using an EV-D68 monoclonal antibody and EV-D68 VLP demonstrated that only animals immunized with EV-D68 VLP generated EV-D68 binding antibody. C. Sandwich ELISA using and EV-A71 monoclonal antibody and EV-A71 VLP demonstrated that only animals immunized with EV-A71 VLP generated EV-A71 binding antibody. D. Indirect Polio ELISA against all three poliovirus serotypes demonstrated that only animals that received IPV generated binding antibody. Data are shown as individual points with mean, error bars indicate standard deviation, n = 10 per group. There were no significant differences between groups (p<0.05) when compared by one-way ANOVA. Diagram in Fig 3A was created with BioRender.com.

We asked whether immunization with IPV or NPEV VLP elicited cross-reactive antibodies in mice. CB6F1 mice (n = 10/group) were immunized at weeks 0, 3, and 11 with 2 μg of either IPV, EV-D68 VLP, or EV-A71 VLP, or both VLPs combined (2 μg of each). All immunogens were adjuvanted with 10% Adjuplex (Fig 3A). Sera were obtained two weeks after each immunization and antibody responses against NPEV VLPs were measured by sandwich ELISA using monoclonal antibodies generated in our lab (S1 and S2 Figs). We observed strong homologous binding antibody responses but no heterologous binding antibodies to NPEV VLPs suggesting that monovalent VLP immunization does not generate cross-reactive NPEV antibodies (Fig 3B and 3C). Binding antibodies to both EV-D68 and EV-A71 VLPs were only present in mice immunized with both VLPs, with no reduction in binding when compared to monovalent vaccination. IPV antibody responses were measured using a commercial polio indirect ELISA kit against all three types of inactivated poliovirus and binding antibodies were only present in mice immunized with IPV (Fig 3D). These results suggest that repeated IPV immunization in mice does not generate cross-reactive antibody responses to NPEVs, and vice versa.

To determine if contaminating host cell proteins in NPEV VLP or IPV preparations confound the interpretation of ELISA results we performed indirect ELISAs with the same mouse sera. Detectable binding antibody responses to both EV-D68 and EV-A71 VLPs were present in sera from animals that received heterologous immunizations (S3–S5 Figs). Taken together these results suggest that immunization with NPEV VLPs or IPV does not result in the generation of cross-reactive antibody responses but induces antibody responses to off-target contaminating host cell proteins, leading to false-positive signals in less stringent assays. Furthermore, our data suggest that indirect ELISA is not sufficient to determine the specificity of NPEV VLP and IPV induced antibody response. Even though cross-reactive responses were not elicited, bivalent immunization elicited responses to both EV-D68 and EV-A71, with no disadvantage of immunizing mice with both VLPs.

### Homologous immunization is necessary to generate enterovirus neutralizing antibodies in mice

**Fig 4:**
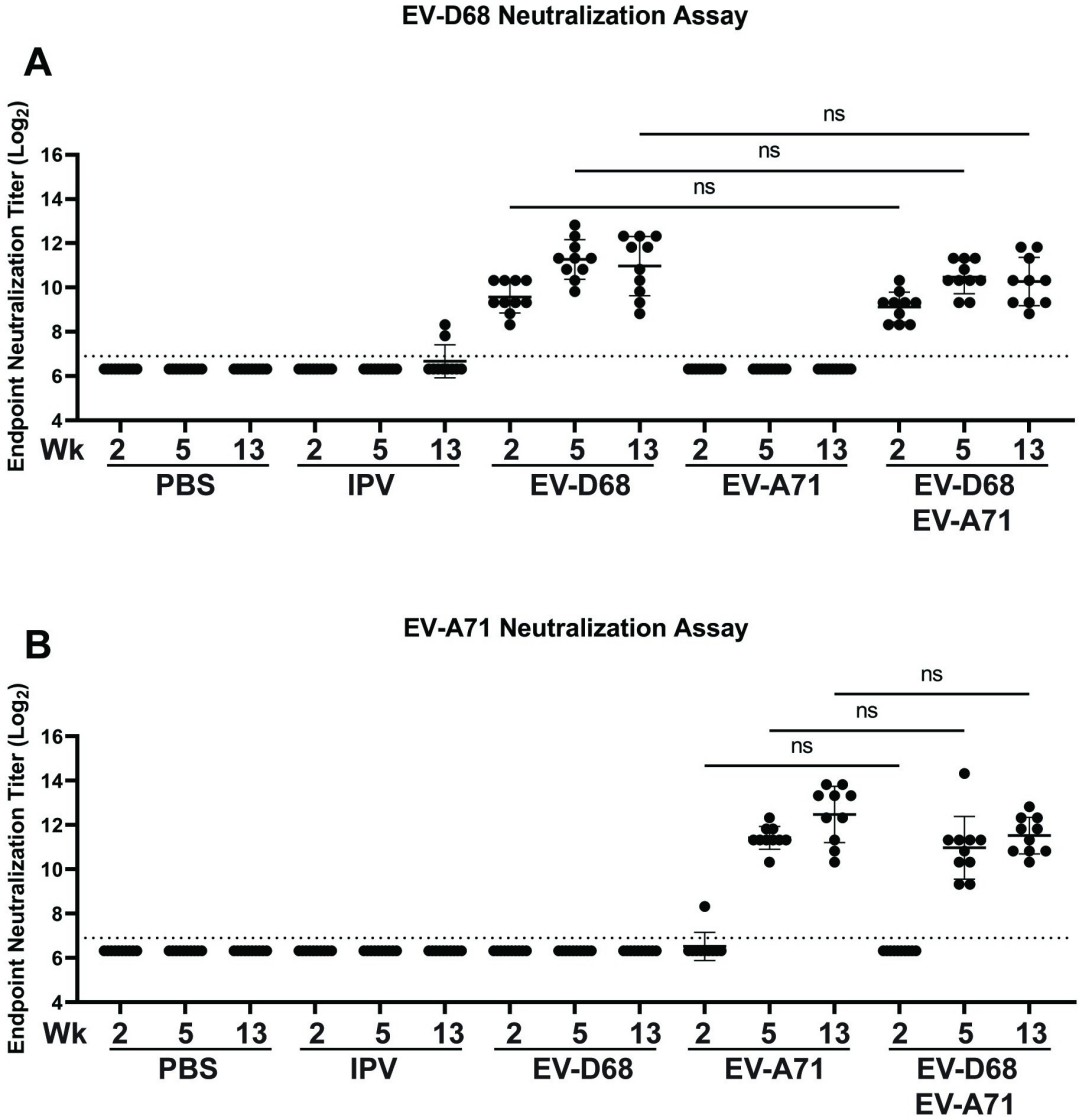
Homologous immunization is necessary to generate enterovirus neutralizing antibodies in mice. Endpoint serum neutralization against EV-D68 (A) and EV-A71 (B) show that immunization with the corresponding VLP is required to generate neutralizing antibodies. We observed similar neutralization titers in groups that receive monovalent or bivalent EV-D68 and EV-A71 VLP formulations. EV-A71 neutralizing antibodies were only detected after a second dose of EV-A71 VLP in both the monovalent and bivalent groups. Data are shown as individual points with mean, error bars indicate standard deviation, n = 10 per group. There were no significant differences between groups (p<0.05) when compared by one-way ANOVA.

We next assessed neutralizing activity against EV-D68 and EV-A71 in endpoint neutralization assays. We observed neutralization against EV-D68 or EV-A71 in groups that received monovalent EV-D68 or EV-A71 VLP, respectively (Fig 4A and 4B). The absence of cross-binding and cross-neutralizing responses indicates that effective vaccination for both EV-D68 and EV-A71 will require the use of both antigens. Repeated immunization with inactivated polio vaccine was insufficient to generate binding or neutralizing antibody to either EV-D68 or EV-A71 VLPs, suggesting that the specificity of polyclonal immunity to enteroviruses is likely to be an impediment to the generation of broad, cross-protective responses using a small number of immunogens. Furthermore, responses to each virus were not reduced when mice were immunized with the combined VLP, demonstrating that a bivalent EV-D68 and EV-A71 VLP vaccine generates potent neutralizing antibodies against both viruses.

### Immunization of baboons with IPV does not induce cross-reactive binding or neutralizing enterovirus antibody responses

**Fig 5:**
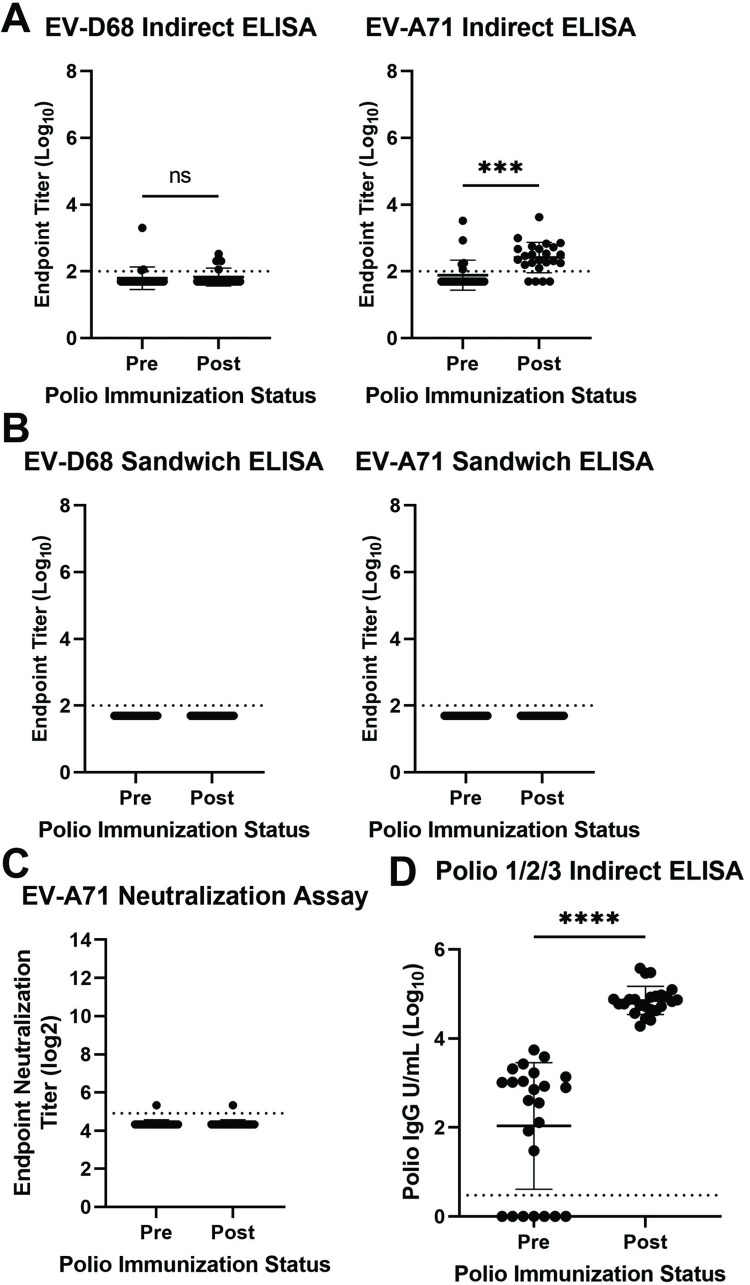
Immunization of baboons with IPV fails to generate cross-reactive neutralizing enterovirus antibody responses. A. Indirect ELISA against EV-D68 or EV-A71 VLP using serum from baboons before and after three doses of IPV. Data are shown as individual points with mean, error bars indicate standard deviation, n = 24. Mean titers were compared by paired t-test with p = 0.6677 for EV-D68 indirect ELISA and p = 0.0002 for EV-A71 indirect ELISA. B. Sandwich ELISA for EV-D68 or EV-A71 VLP using serum from baboons before and after three doses of IPV. Data are shown as individual points with mean, error bars indicate standard deviation, n = 24. C. EV-A71 endpoint neutralization assay using serum samples from baboons before and after IPV immunization. D. Some baboons possessed IPV binding antibodies before immunization, but all animals exhibited a robust boost response following IPV immunization. Mean titers were compared by paired t-test with p = <0.0001. Data are shown as individual points with mean, error bars indicate standard deviation, n = 24. Asterisks indicate p value with *** indicating p < 0.001 and **** indicating p < 0.0001, ns indicates a p value > 0.05 determined by paired t-test.

To further confirm the lack of vaccine-induced cross-reactive antibody responses in an animal species more closely related to humans than mice, we next asked whether polio vaccination in baboons conferred binding or neutralizing responses to EV-D68 or EV-A71 VLPs or virus. To address this question in animals with no history of infection with either NPEV, we obtained serum samples from baboons vaccinated with either pentacel [38] (which contains IPV) or IPOL. Sera obtained before their first dose and two weeks after their last dose were used for our studies. First, we measured EV-D68 and EV-A71 binding antibodies by indirect ELISA. We observed no binding antibodies against EV-D68 VLP before or after immunization with pentacel or IPOL (Figs 5A and S6). We did observe an increase in EV-A71 VLP binding antibodies after vaccination, but this may be due to detection of non-specific proteins by indirect ELISA (S3 Fig). When we measured EV-D68 and EV-A71 binding antibody responses in baboons by sandwich ELISA, both were undetectable (Fig 5B). These results support our hypothesis that cross-reactive antibody responses detected by indirect ELISA are generated against contaminating host cell proteins. EV-A71 endpoint neutralization assay further confirmed that the immune sera did not neutralize EV-A71 (Fig 5C). We detected poliovirus binding antibodies in most baboons before vaccination, but poliovirus antibody responses were boosted in all animals following completion of the vaccine series (Fig 5D). Baboons received their first dose at ages five to seven months and preimmunization serum was obtained thirty days before their first dose. It is possible that these poliovirus antibodies are maternally derived, or due to background for baboon samples in the commercial polio ELISA kit. Our results demonstrate that immunization of baboons with IPV is not sufficient to generate binding and neutralizing antibody responses against EV-D68 or EV-A71.

## Discussion

The discovery of enterovirus-specific cross-reactive antibodies in hyperimmune animals and humans may call into question the need for vaccine development for emerging and re-emerging enteroviruses. Yet, these antibodies exhibit limited breadth, and it is unclear how easily they could be elicited by typical two or three-dose vaccine regimens [31]. Many of the antibodies identified also bind peptides rather than conformational epitopes, where the most potent neutralizing antibodies bind [32]. Here, we demonstrate that three vaccinations with inactivated polio vaccines or VLP vaccine candidates for EV-D68 and EV-A71 fail to elicit cross-reactive responses in mice and baboons, supporting the notion that homologous and/or multivalent vaccines will be required to protect against human disease due to these viruses.

The Centers for Disease Control and Prevention in the United States recommends four doses of inactivated polio vaccine, three before 18 months of age [39]. Despite polio vaccination rates exceeding 90% in the US, EV-D68 causes biennial outbreaks of respiratory disease and has been causally linked to AFM [5,40]. Likewise, polio vaccine uptake for 1-year-olds in China has been 99% since 2008, yet China approved and deployed three highly effective inactivated EV-A71 vaccines in 2016 to combat neurologic consequences of hand foot and mouth disease caused by EV-A71 [41,42]. An epidemiological modeling study has suggested that infection with closely related enterovirus species such as EV-A71 and CV-A16 can induce cross-protective immunity in humans [43,44]. However, a recent surveillance and epidemiology study reported that coxsackievirus A6 has replaced EV-A71 as the major cause of hand, foot and mouth disease in Nanchang China following the deployment of an EV-A71 vaccine, suggesting that vaccination against one enterovirus may lead to replacement by another [45]. The implication of these real-world observations and our studies is that the most effective strategy for combating severe disease outcomes of enterovirus infections in human populations is by vaccination against the corresponding enterovirus of concern or a multivalent strategy.

As studies in humans are complicated by unknown exposure history, evaluation of immune responses to enterovirus vaccines in animals may be complicated by contaminants present in vaccine preparations. This is due to the complexity of purification without affinity tags which can result in co-purification with other proteins. We found that apparent cross-reactive responses measured using indirect ELISA were likely specific to contaminating host-cell proteins. The use of monoclonal antibodies and a sandwich ELISA removed the signal from these off-target responses. Similar off-target responses may account for differences between our results and a study that observed cross-reactive responses between polio, EV-D68, and EV-A71 [31]. There are other notable methodological differences as that study by Rosenfeld et al. [31] included five intraperitoneal inoculations with live virus emulsified in complete Freund’s adjuvant. In our hands, sera from mice immunized three times with IPV did not bind or neutralize EV-D68 or EV-A71 despite having a robust IPV antibody response. Similarly, serum obtained from IPV vaccinated baboons with a high titer of IPV binding antibodies was incapable of neutralizing either EV-D68 or EV-A71, suggesting that this observation is not limited by an inbred mouse model and may be applicable to humans.

Our findings and methods are not without limitations. We used inactivated poliovirus vaccine and VLPs based on EV-D68 and EV-A71 as immunogens, rather than live viruses, which are known to have slightly altered conformations compared to a mature virion [20,25]. However, both EV-D68 and EV-A71 VLPs have been shown to be effective at inducing neutralizing antibody responses in both mice and nonhuman primates [17,46,47]. Furthermore, IPV immunization is standard for protection against poliomyelitis in the United States and Europe and has been shown to induce strong poliovirus neutralizing antibodies in both mice and nonhuman primates [48,49]. Based on these observations we do not expect that failure to generate cross-reactive enterovirus antibodies in this study is the result of using IPV or VLPs as immunogens.

As done for the three serotypes of polio, multivalent vaccines comprising several antigens may be necessary to extend protection across distinct enterovirus species. A bivalent VLP based vaccine was shown to protect mice against infection from enterovirus EV-A71 and CV-A16 [46]. Our results confirm that multivalency does not adversely affect EV-D68 or EV-A71 responses and highlight the feasibility of this approach for NPEVs. Similar approaches have been tested preclinically for human rhinoviruses (HRV) with a 50-valent formulation exhibiting efficacy in nonhuman primates [50,51]. Several approved vaccines for other targets, including human papilloma virus (HPV) also use this multivalent approach to increase the breadth of protection [52].

In summary, our data demonstrate that conventional vaccination strategies for poliovirus, EV-A71, or EV-D68 do not elicit cross-reactive antibodies to the heterologous viruses. We conclude that multivalent NPEV vaccines to combat existing and emerging enteroviruses associated with human morbidity and mortality is a feasible approach.

## Materials and methods

### Ethics statement

Mouse studies followed the *Guide for the Care and Use of Laboratory Animals* from the National Institutes of Health and the Animal Welfare Act code of Federal Regulations. Animal studies were approved by the Vaccine Research Center Animal Care and Use Committee under protocol VRC-21-0920. All nonhuman primate procedures were performed at Michael E Keeling Center for Comparative Medicine and Research at the University of Texas MD Anderson Cancer Center (Bastrop, TX) accredited by the Association for Assessment and Accreditation of Laboratory Animal Care International, in accordance with protocols approved by the Institutional Animal Care and Use Committee at the University of Texas MD Anderson Cancer Center and the principles outlined in the Guide for the Care and Use of Laboratory Animals [53] by the Institute for Laboratory Animal Resources, National Research Council.

### Enterovirus capsid conservation analysis

To analyze enterovirus capsid sequence conservation, we obtained the structure and amino acid sequence of an EV-D68 B1 isolate from the protein data bank (PDB: 6CRR) and performed a BLAST analysis with Uniprot (https://www.uniprot.org/blast) and selected 40 of the top scoring sequences, which were aligned using Clustal Omega [54]. The structure and multiple sequence alignment were loaded into ChimeraX software which was used to calculate sequence conservation score using the AL2CO algorithm and color the capsid residues [37,55]. The multiple sequence alignment was imported into Geneious prime and used to generate a phylogenetic tree using the Geneious tree builder.

### Immunogen preparation

EV-D68 and EV-A71 VLPs were prepared as previously described [17]. Briefly, capsid protein sequences and 3CD protease gene sequences from EV-D68 B3 isolate 23209 and EV-A71 23092 were codon optimized and cloned into expression vectors. Plasmid DNA was used to transfect Expi293 cells following the manufacturer’s instructions. Four to five days after transfection, cell cultures were harvested and frozen at -80C. Cultures were thawed and centrifuged at 2700 xg for 20 min to pellet cell debris and filtered through a 0.45 um filter. Supernatant was then centrifuged over a 20% w/v sucrose cushion in Tris-NaCl-EDTA (TNE) buffer at 20,000 rpm and 4C for 2.5 hours using a Beckman SW-32Ti rotor. Sucrose cushion pellets were resuspended in TNE overnight and loaded onto a 15–45% sucrose gradient and centrifuged at 17,000 rpm and 4C for 18 hours using a Beckman SW-41Ti rotor. Gradients were fractionated and the presence of VLP was confirmed by Pierce BCA protein assay and SDS-polyacrylamide gel electrophoresis (SDS-PAGE) under reducing conditions. VLP-containing fractions were pooled, and buffer exchanged into sterile phosphate buffered saline (PBS) using a Millipore amicon 100,000 kDa cutoff centrifugal concentrator. VLP concentration was measured by Pierce BCA protein assay and purity was assessed by SDS-PAGE. Commercial inactivated poliovirus vaccine (IPOL, Sanofi-Pasteur) was obtained in 10 mL vials. Vials were pooled and concentrated using a Millipore amicon 100,000 kDa cutoff centrifugal concentrator and protein content was measured by Pierce BCA assay. Purity was assessed by SDS-PAGE. For the assessment of purity of VLPs and viral stocks 5 μg of material was loaded onto an SDS-PAGE gel and run under reducing conditions. The presence of VLP or IPV particles was confirmed by transmission electron microscopy. Immunogens were aliquoted and stored at -80C until use for animal studies.

### Cells and viruses

Enterovirus D68 (23087) and enterovirus A71 (23092) isolates were obtained from BEI resources and cultured in RD cells (American Type Culture Collection, CCL-136) grown in Dulbecco’s modified Eagle’s medium (Invitrogen, Carlsbad, CA) supplemented with 10% fetal bovine serum and 1% penicillin/streptomycin as described [17]. Confluent 175 cm^2^ flasks were infected with either virus at a multiplicity of infection (MOI) of 0.01 and were incubated at 33°C for EV-D68 or 37°C for EV-A71. Flasks were monitored for cytopathic effect (CPE) and culture media was harvested when more than 80% of the cells exhibited CPE. Culture media was frozen at -80°C and thawed then centrifuged at 2700 xg for 20 minutes and filtered through at 0.45 μm filter to remove cell debris. Viral titer was assessed by TCID_50_ endpoint titration using the Spearman-Karber method and RD cells [56]. This preparation was assessed for host-cell proteins by SDS-PAGE and used to generate neutralization stocks for serum neutralization assays.

### Vaccination of animals

CB6F1 mice (Jackson Laboratory) were used for all mouse studies. VLPs or IPV were mixed with 10% Adjuplex in PBS and each animal received a 2 μg dose of VLP or IPV intramuscularly with a half dose in each inner thigh. Mice receiving bivalent vaccination received 2 μg of each VLP. Booster vaccinations were given at weeks 3 and 11 and serum was sampled two weeks after each immunization. Serum from each bleed was stored at -20C until use. Baboons (*Papio anubis*) were vaccinated with Pentacel (Sanofi Pasteur Limited) or IPOL (Sanofi Pasteur, SA) three times two months apart. Sera were collected one month before the first vaccination and on day 17 post third vaccination.

### ELISA

For sandwich ELISA MaxiSorp 384-well plates (Thermo Fisher Scientific) were coated with 25 μL of 1 μg/mL EV-D68 or EV-A71 monoclonal antibody generated in our laboratory in PBS overnight at 4C. The following day, plates were washed three times with 100 μL per well of PBS containing 0.05% (v/v) Tween 20 (PBST) and blocked for 1 hour at room temperature with blocking buffer, PBST containing 5% non-fat dry milk (NFDM, Milipore). Plates were washed again as described and incubated with 25 μL of EV-D68 or EV-A71 VLP at 1 μg/mL in PBS for 1 hour at room temperature and then washed again as described. Mouse serum was diluted 1:100 in blocking buffer and serially diluted 1:10 in blocking buffer and 20 μL of diluted serum transferred to the coated plates and incubated for 1 hour at room temperature. Plates were washed as described and incubated with 25 μL per well of horseradish peroxidase conjugated goat-anti-mouse-IgG at a 1:6000 dilution in blocking buffer for 1 hour at room temperature. Plates were washed as described and developed with 50 μL per well of KPL SureBlue 1-component TMB peroxidase substrate for 10 minutes at room temperature. Development was stopped by the addition of 50 μL of 1-N sulfuric acid and absorbance at 450 and 650 nm was read using a Bio-Tek plate reader. For analysis the 650 nm reading was subtracted from the 450 nm reading. Serum dilutions were log-transformed, and absorbance values were fit by non-linear regression using GraphPad Prism 9.3 software. Endpoint titer was determined as the serum dilution that reached a 450 nm absorbance signal of 0.15, more than three-fold higher than the average of negative control wells. For indirect ELISA, plates were coated with VLP at 1 μg/mL in PBS overnight at 4C and steps proceeded as described for sandwich ELISA after blocking and serum incubation. Each serum sample was tested in duplicate technical replicates. Poliovirus ELISA was performed using a kit from Alpha Diagnostic International (Catalog # 970-120-PMG for mouse and 970-150-PMG for monkey) following the manufacturer’s instructions. Polio 1/2/3 IgG titers were determined using a standard curve and log transformed before graphing using GraphPad Prism 9.3 software. IPV immunized samples were measured at 1:100, 1:500, 1:2500 and 1:12500 serum dilutions while VLP or PBS immunized samples were only measured at 1:100 serum dilution. All samples were measured in duplicate technical replicates. Mean titers of groups with mean titers above the limit of detection were compared by one-way ANOVA and Šídák’s multiple comparisons test.

### Neutralization assay

Serum samples were heat-inactivated at 56C for 30 minutes and then diluted in two-fold serial dilutions in DMEM containing 1% penicillin/streptomycin in untreated U-bottom 96-well plates. An equal volume of working virus neutralization stock containing 200 TICD_50_ of EV-D68 or EV-A71 virus was added and serum-virus mixtures were incubated at 33C (for EV-D68) or 37C (for EV-A71) for 1 hour in a cell culture incubator. After incubation serum-virus mixtures were added to 96-well plates containing 95% confluent monolayers of RD cells and returned to the incubator and the indicated temperature for each virus and incubated for 4 days for EV-A71 and 5 days for EV-D68. After incubation plates were scored for the presence of cytopathic effect and fixed and stained with ExCellPlus fixative (StatLab) containing crystal violet. The endpoint neutralization titer is defined at the highest serum dilution that completely blocks the development of viral CPE. Each serum sample was tested in duplicate technical replicates. Mean titers of groups with mean titers above the limit of detection were compared by one-way ANOVA and Šídák’s multiple comparisons test.

## Supporting information

S1 Fig Sandwich ELISA to detect EV-D68 VLP binding antibodies. Non-linear regression analysis was used to fit ELISA binding data measured by optical density at 450 nm. Endpoint titers were determined as described in materials and methods.
(TIF)

S2 Fig Sandwich ELISA to detect EV-A71 VLP binding antibodies. Non-linear regression analysis was used to fit ELISA binding data measured by optical density at 450 nm. Endpoint titers were determined as described in materials and methods.
(TIF)

S3 Fig Indirect enterovirus VLP ELISA identifies non-specific contaminating host-cell protein antibodies. Endpoint binding antibody titers against EV-D68 (A) or EV-A71 (B) measured by indirect ELISA. Data are shown as mean titer with error bars indicating standard deviation, n = 10 animals per group. A larger proportion of animals exhibit heterologous binding antibodies when measured by indirect ELISA compared to sandwich ELISA.
(TIF)

S4 Fig Indirect ELISA to detect EV-D68 VLP binding antibodies. Non-linear regression analysis was used to fit ELISA binding data measured by optical density at 450 nm. Endpoint titers were determined as described in materials and methods.
(TIF)

S5 Fig Indirect ELISA to detect EV-A71 VLP binding antibodies. Non-linear regression analysis was used to fit ELISA binding data measured by optical density at 450 nm. Endpoint titers were determined as described in materials and methods.
(TIF)

S6 Fig Indirect and sandwich ELISA to detect EV-D68 and EV-A71 VLP binding antibodies in polio immunized baboons. Indirect ELISA (top) and sandwich ELISA (bottom). Non-linear regression analysis was used to fit ELISA binding data measured by optical density at 450 nm. Endpoint titers were determined as described in materials and methods.
(TIF)

S1 Data Raw data for all ELISA and neutralization assays. ELISA data is presented as absorbance value at 450 nm and neutralization assay data is presented as endpoint dilution titer.
(XLSX)
