# Supplementary figures and images for: Enterovirus virus-like-particle and inactivated poliovirus vaccines do not elicit substantive cross-reactive antibody responses
Source: PLoS Pathog. 2024 Apr 25;20(4):e1012159. doi: 10.1371/journal.ppat.1012159 (PMC11045126; doi:10.1371/journal.ppat.1012159)

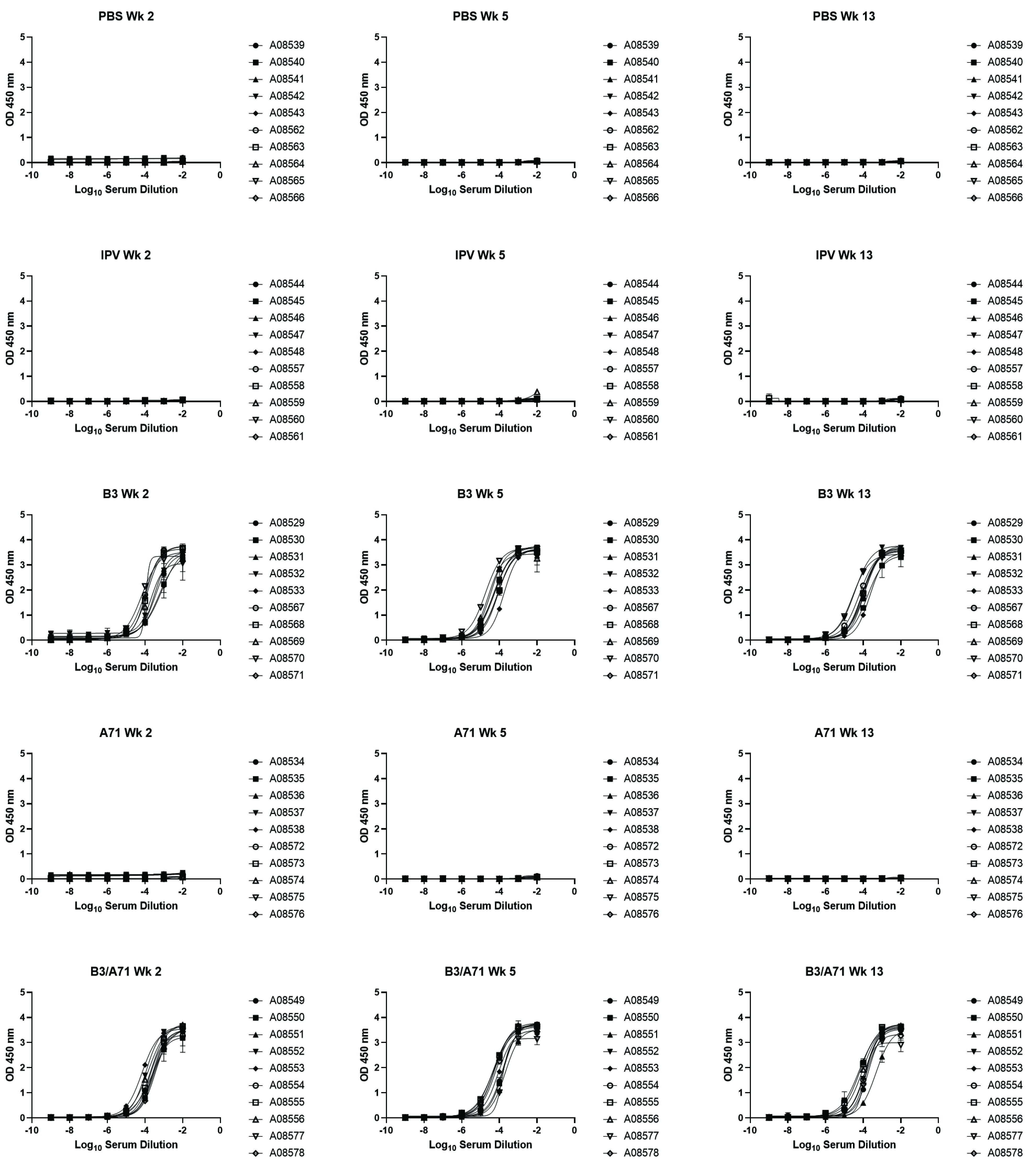

Supplement: S1 Fig — Non-linear regression analysis was used to fit ELISA binding data measured by optical density at 450 nm. Endpoint titers were determined as described in materials and methods. (TIF) [file ppat.1012159.s001.tif]

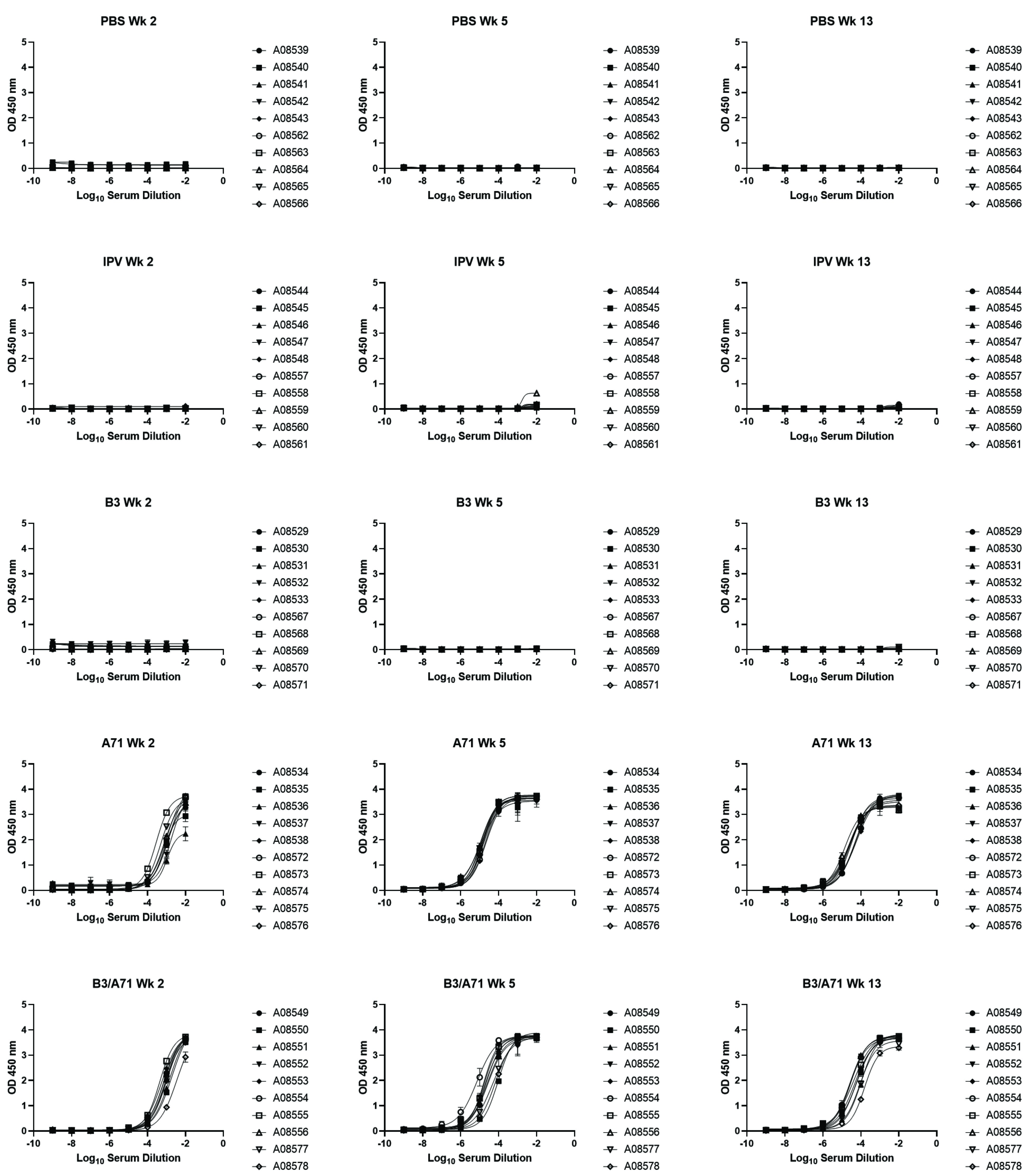

Supplement: S2 Fig — Non-linear regression analysis was used to fit ELISA binding data measured by optical density at 450 nm. Endpoint titers were determined as described in materials and methods. (TIF) [file ppat.1012159.s002.tif]

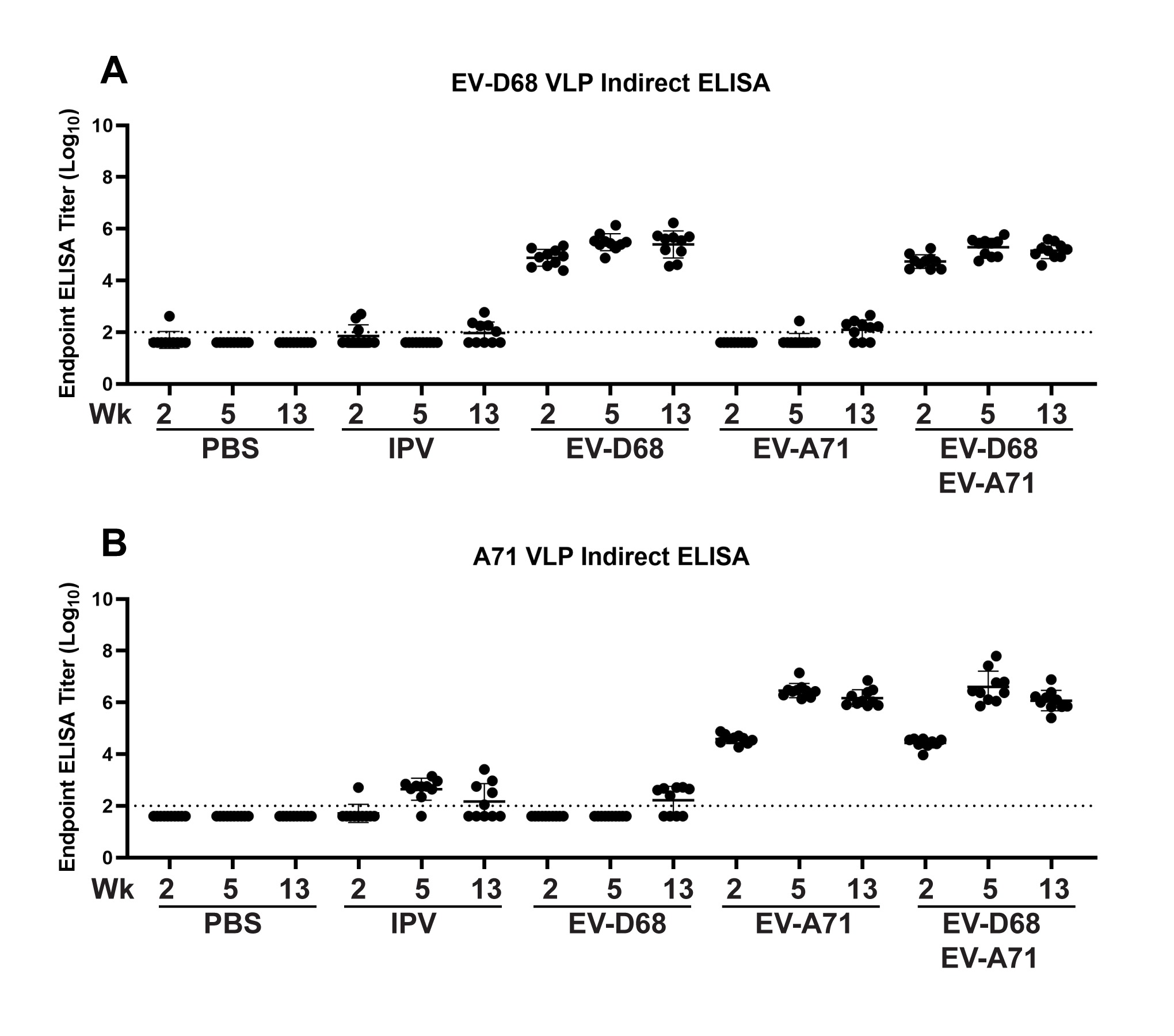

Supplement: S3 Fig — Endpoint binding antibody titers against EV-D68 (A) or EV-A71 (B) measured by indirect ELISA. Data are shown as mean titer with error bars indicating standard deviation, n = 10 animals per group. A larger proportion of animals exhibit heterologous binding antibodies when measured by indirect ELISA compared to sandwich ELISA. (TIF) [file ppat.1012159.s003.tif]

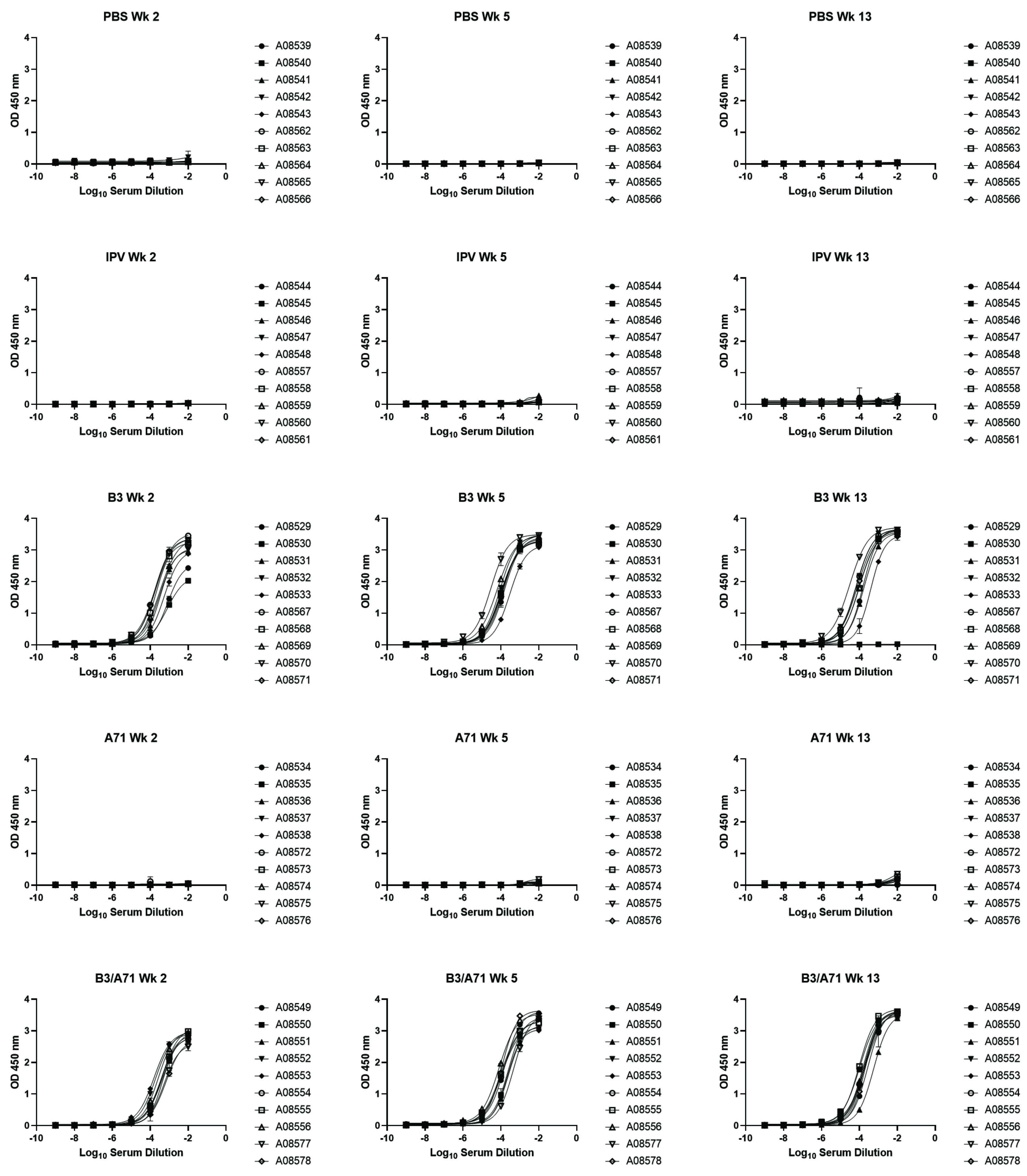

Supplement: S4 Fig — Non-linear regression analysis was used to fit ELISA binding data measured by optical density at 450 nm. Endpoint titers were determined as described in materials and methods. (TIF) [file ppat.1012159.s004.tif]

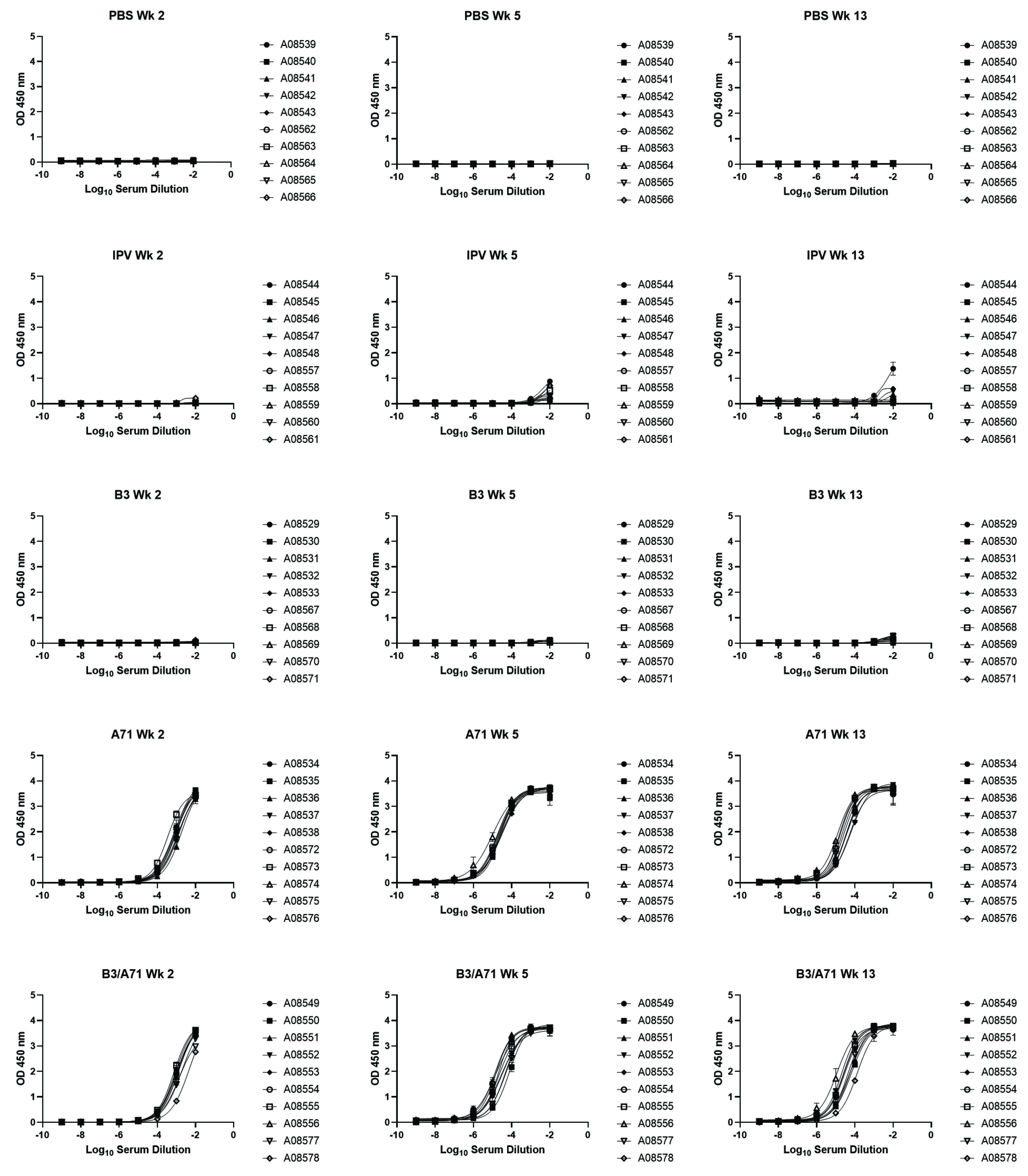

Supplement: S5 Fig — Non-linear regression analysis was used to fit ELISA binding data measured by optical density at 450 nm. Endpoint titers were determined as described in materials and methods. (TIF) [file ppat.1012159.s005.tif]

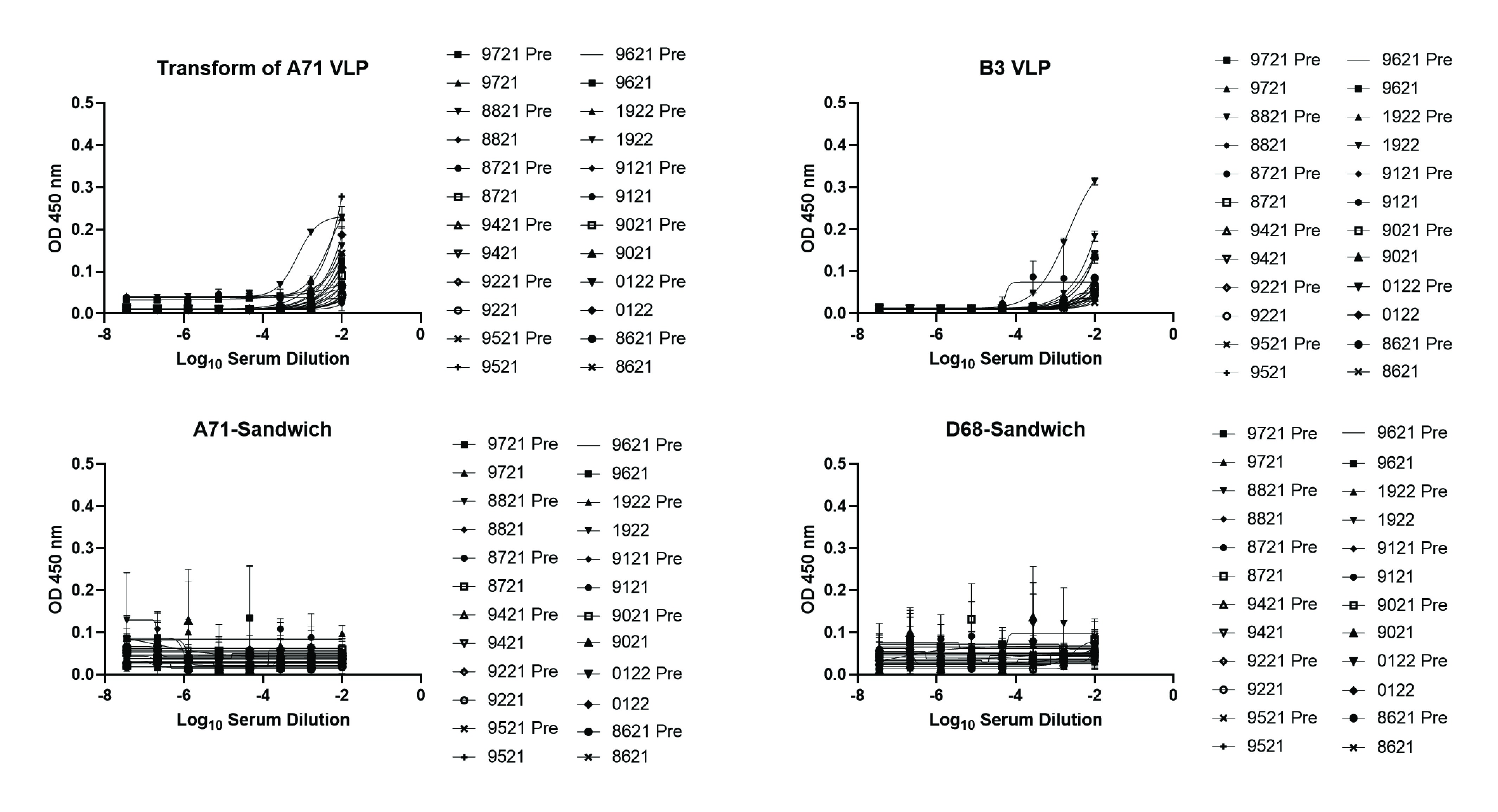

Supplement: S6 Fig — Indirect ELISA (top) and sandwich ELISA (bottom). Non-linear regression analysis was used to fit ELISA binding data measured by optical density at 450 nm. Endpoint titers were determined as described in materials and methods. (TIF) [file ppat.1012159.s006.tif]
